# Association of Renal Hyperfiltration with Incidence of New-Onset Diabetes Mellitus: A Nationwide Cohort Study

**DOI:** 10.3390/jcm13175267

**Published:** 2024-09-05

**Authors:** Min-Ju Kim, Min Kyoung Kang, Ye-Seon Hong, Gwang Hyun Leem, Tae-Jin Song

**Affiliations:** 1 Department of Neurology, Seoul Hospital, Ewha Womans University College of Medicine, Seoul 07804, Republic of Korea; lisa0926@naver.com (M.-J.K.); eiri616@hanmail.net (M.K.K.); 2 Department of Physiology, Ewha Womans University College of Medicine, Seoul 07804, Republic of Korea; 02yesun@naver.com; 3 Department of Convergence Medicine, Seoul Hospital, Ewha Womans University College of Medicine, Seoul 07804, Republic of Korea; shalomlkh@ewha.ac.kr

**Keywords:** diabetes mellitus, renal function, high estimated glomerular filtration rate, glomerular hyperfiltration, population study

## Abstract

**Background and Objectives:** While the connection between decreased kidney function and diabetes mellitus (DM) is commonly acknowledged, there is insufficient research examining the relationship between higher-than-normal estimated glomerular filtration rate (eGFR) and the incidence risk of new-onset DM. Our research aimed to explore the relationship between an eGFR and the incidence risk of new-onset DM in the Korean general population through a nationwide longitudinal study. **Methods**: This research employed the cohort records of the National Health Insurance Service in Korea, analyzing records from 2,294,358 individuals between the ages of 20 and 79 who underwent health check-ups between 2010 and 2011. The eGFR levels from the Chronic Kidney Disease Epidemiology Collaboration (CKD-EPI) equation were used to assess the renal function. New-onset DM was defined as two or more claims with the International Classification of Diseases-10 classification codes E10 to E14, being prescribed any medication for lowering blood glucose, or having a record of fasting plasma glucose levels of ≥126 mg/dL from a health examination after the index date. **Results:** The mean age of subjects was 47.34 ± 13.76 years. The 150,813 (6.57%) new-onset DM cases were identified over a median follow-up of 9.63 years. In the multivariable Cox regression analysis, in comparison with the 5th decile, the 10th (≥114.12 mL/min/1.73 m^2^) (hazard ratio (HR): 0.52, 95% confidence interval (CI) (0.50–0.54), *p* < 0.001) eGFR decile was significantly associated with a decreased incidence of new-onset DM. Moreover, eGFR >120 mL/min/1.73 m^2^ was associated with a reduced risk of new-onset DM (HR: 0.40, 95% CI (0.39–0.42), *p* < 0.001). These results were consistent regardless of the presence of impaired glucose tolerance, age, or obesity. **Conclusion:** Our study showed higher-than-normal eGFR levels were associated with a lower risk of incidence for new-onset DM regardless of the presence of impaired glucose tolerance, age, or obesity. In general population, higher-than-normal eGFR may be associated with a lower risk of incidence of new-onset DM.

## 1. Introduction

Impaired kidney function is associated with cerebrovascular and cardiovascular diseases or their risk factors such as stroke, atrial fibrillation, myocardial infarction, and heart failure [1,2]. A common indicator of chronic kidney disease (CKD) is a diminished estimated glomerular filtration rate (eGFR), and decreased eGFR levels have a significant correlation with the likelihood of developing cardiovascular disease [1,3]. Besides low eGFR, an unusually higher-than-normal eGFR can also be associated with several health issues. Although a higher-than-normal eGFR is typically seen as a regular physiological condition or better renal function, it might signal underlying abnormal kidney function. For instance, it suggests the presence of preclinical kidney disease in diabetic patients or could indicate early signs of damage to the filtering units of the kidney in those with high blood pressure or hypertension [4,5,6]. Higher-than-normal eGFR is closely linked with hypertension and obesity, which could contribute to the occurrence of cerebrovascular or cardiovascular events [4,5].

Over the last hundred years, diabetes mellitus (DM) has emerged as a major global health crisis affecting both Western and Eastern countries [7,8]. DM is not only associated with well-known microvascular complications like neuropathy, nephropathy, and retinopathy, but also with a growing incidence of macrovascular complications, such as those affecting the carotid, coronary, cerebral, and peripheral arteries [9]. To prevent the onset of DM, several strategies have been recommended. These include maintaining a healthy body weight and waist circumference, engaging in regular physical activity, adopting healthy eating habits, and regular health check-ups to identify potential risk factors [10]. Despite these measures, there remains a lack of effective drugs and preventive approaches specifically targeting the emergence of new-onset DM.

Because higher-than-normal eGFR is often seen as an indicator of fine renal function, a population displaying this elevated eGFR may potentially experience a decreased likelihood of developing DM. On the other hand, if a higher-than-normal eGFR is an early sign of kidney disease, it might imply a greater risk of impaired glucose tolerance or ultimately DM [11]. Up to now, there has been limited research on whether a higher-than-normal eGFR may be related to the onset of DM. Our research sought to examine the link between higher-than-normal eGFR and the incidence risk of new-onset DM in the Korean general population through a nationwide longitudinal study.

## 2. Materials and Methods

### 2.1. Data Source

This study sourced its data from the National Health Insurance Service-Health Screening Cohort (NHIS-HEALS) database, a subset of the Korean NHIS. The NHIS, a government program, provides health insurance to nearly 97% of the Korean population. The Medical Aid program, an affiliate of the NHIS, attends to the 3% of the population not covered by the NHIS [12,13].

The NHIS encourages participants to undergo standardized health check-ups every two years to aid in early identification and prevention of diseases. The NHIS-HEALS database collects a range of information, including demographic details, socioeconomic background, health screening results, recorded diagnoses, and treatment specifics. These screenings involve assessments like height, weight, blood pressure, lab tests, and evaluations of lifestyle behaviors [14].

### 2.2. Study Population

**Figure 1:**
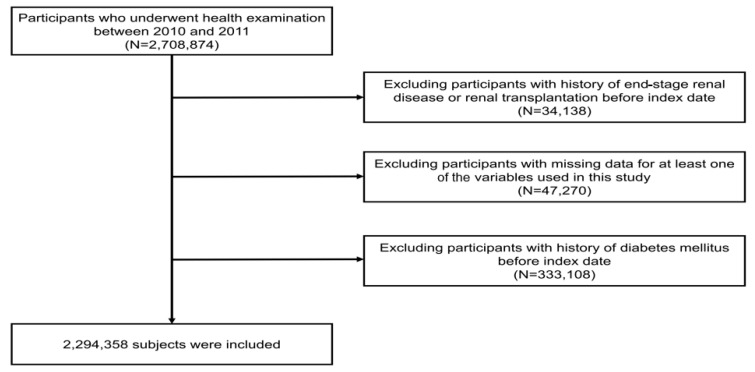
Diagram illustrating the process of selecting participants for the study.

This study’s cohort from the NHIS-HEALS database included 2,708,874 individuals aged between 20 and 79 who took part in health screenings from 2010 to 2011 (under the dataset identifier: NHIS-2022-01-313) [13,14,15]. From this group, we excluded those who had previously been diagnosed with end-stage renal disease (ESRD) before the research’s starting point, totaling 34,138 individuals. Moreover, we excluded 47,270 individuals who had missing demographic or laboratory data. Additionally, 333,108 individuals with a history of hypertension were excluded at the commencement of the study. Finally, this study’s analysis involved a sample of 2,294,358 participants (Figure 1).

### 2.3. Definitions and Variables

The starting point for tracking each participant’s outcome, referred to as the index date, was established based on the date of their health evaluation. To determine the eGFR, serum creatinine levels from the health check-up were used along with the formulas provided by the Chronic Kidney Disease Epidemiology Collaboration (CKD-EPI) (Supplementary Materials) [16]. Baseline characteristics, including age, gender, body mass index, waist circumference, and household income, were evaluated on the index date [17,18]. Through questionnaires, details on habits like smoking, drinking alcohol, and regular exercise were collected. Participants’ smoking habits were classified as non-smoking, past smoking, or current smoking. Both alcohol intake and consistent physical activity were noted in terms of how often they occurred weekly. Proteinuria was confirmed if the urine dipstick test showed a result of ≥+1. Comorbidities, including hypertension, dyslipidemia, heart failure, myocardial infarction, valvular heart disease, cardiomyopathy, hyperthyroidism, congenital heart disease, and Charlson comorbidity index, were recognized using specific criteria from January 2009 to the index date (Supplementary Materials). Diagnostic codes were categorized according to the International Classification of Diseases (ICD)-10, following methodologies from prior research [19,20]. The criteria for identifying new-onset DM involved recognizing it as a primary or secondary condition, as per the ICD-10 classification codes E10 to E14. This diagnosis was established by having at least one annual claim for both outpatient visits and hospital admissions, along with records of being prescribed any medication for lowering blood sugar. Additionally, a diagnosis could be made if there was at least one record of fasting plasma glucose levels equal to or exceeding 126 mg/dL, as per the data from the NHIS-HEALS, prior to the index date [13,20,21].

### 2.4. Statistical Analysis

The data results are presented either in the form of mean ± standard deviation or expressed as numbers and percentages. To investigate the association between eGFR and the incidence of new-onset DM, participants were stratified into eGFR deciles or classified following eGFR ranges (<30, 30–60, 60–90, 90–120, and >120 mL/min/1.73 m^2^). The reference group consisted of the 5th decile and the range from 60 to 90 mL/min/1.73 m^2^. The examination of this relationship utilized Kaplan–Meier survival curves, and distinctions between eGFR deciles and ranges were assessed through log-rank tests. Hazard ratios (HR) and 95% confidence intervals (CIs) for the correlation between eGFR and the incidence of new-onset DM were calculated using Cox proportional hazard models. Multivariable regression analysis was employed to adjust for potential confounding factors, including variables such as sex, age, body mass index, waist circumference, income levels, smoking, alcohol consumption, regular physical activity, proteinuria, hypertension, diabetes mellitus, dyslipidemia, heart failure, myocardial infarction, valvular heart disease, cardiomyopathy, hyperthyroidism, congenital heart disease, and Charlson comorbidity index. For the sensitivity analysis, further analysis was performed in a population with impaired glucose tolerance (at least one or more times noted with fasting glucose level as 100 to 125 mg without DM). In the context of a sensitivity analysis, an additional assessment was conducted by calculating eGFR levels using the Modification of Diet in Renal Disease (MDRD) study equation [22]. Subgroup analyses were conducted, taking into account age and body mass index, as these variables are closely linked to eGFR levels. Statistical analyses were performed using SAS software (version 9.2, SAS Institute, Cary, NC, USA), and significance was established with a *p*-value of less than 0.05.

## 3. Results

The mean age of subjects was 47.34 ± 13.76 years with men making up 50.50% of the subjects. The prevalence of current smokers, hypertension, and dyslipidemia was 23.74%, 13.16%, and 10.80%, respectively. In terms of eGFR categories, the distribution of individuals with eGFR levels <30, 30–60, 60–90 (used as the reference), 90–120, and >120 mL/min/1.73 m^2^ was 0.06%, 3.31%, 43.83%, 48.24%, and 4.56%, respectively (Table 1).

Considering higher-than-normal eGFR, individuals with eGFR > 120 mL/min/1.73 m^2^ appeared to have a lower mean BMI and smaller waist circumferences and be younger in age in comparison to the other groups (Supplementary Table S1). Moreover, a lower frequency of proteinuria, hypertension, and dyslipidemia was noted in individuals with eGFR > 120 mL/min/1.73 m^2^ (Supplementary Table S1).

Over a median follow-up period of 9.63 years (interquartile range: 9.14–10.12 years), 150,813 (6.57%) new-onset DM cases were identified. Kaplan–Meier survival analysis demonstrated a significant association between the decreased risk of new-onset DM and elevated eGFR in the 10th decile (≥114.12 mL/min/1.73 m^2^) (*p* < 0.001), and higher-than-normal eGFR levels (>120 mL/min/1.73 m^2^) (*p*< 0.001). Considering the range of eGFR, eGFR > 120 mL/min/1.73 m^2^ was also associated with decreased risk of new-onset DM (Supplementary Figure S1).

**Figure 2:**
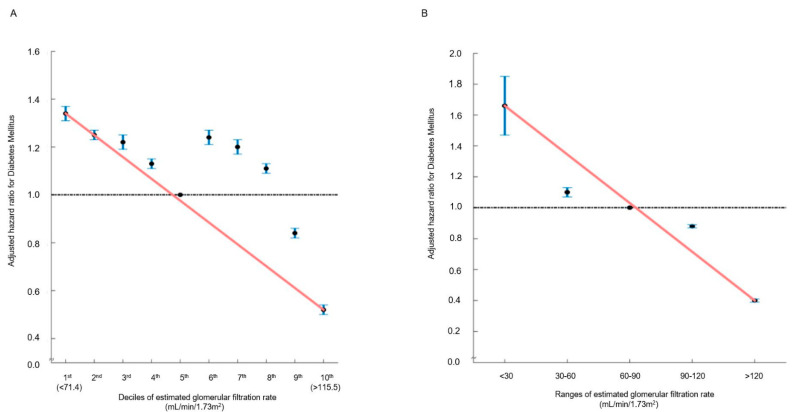
Hazard ratios for new-onset DM determined by estimated glomerular filtration rate ((**A**): deciles; (**B**): ranges). The hazard ratios, depicting the correlation between renal function and the incidence of new-onset DM, are presented either by (**A**) decile groups or (**B**) eGFR ranges. The solid blue line illustrates the multivariate-adjusted hazard ratios with corresponding 95% confidence intervals for each group, while the dashed lines denote a hazard ratio of 1. The red line signifies restricted cubic spline curves. The hazard ratios were calculated using the multivariable Cox model outlined in Table 2.

In the multivariable analysis, in comparison with the 5th decile, the 9th (106.46–114.02 mL/min/1.73 m^2^) (HR: 0.84, 95% CI (0.82–0.86), *p* < 0.001) and 10th (≥114.12 mL/min/1.73 m^2^) (HR: 0.52, 95% CI (0.50–0.54), *p* < 0.001) eGFR deciles were significantly associated with a decreased incidence of new-onset DM (Table 2 and Table S2). Moreover, eGFR > 120 mL/min/1.73 m^2^ was associated with a reduced risk of new-onset DM (HR: 0.40, 95% CI (0.39–0.42), *p* < 0.001) (Table 2 and Table S3). Both in decile and range groups, the hazard ratio plot illustrated a decrease in the hazard ratio for new-onset DM as eGFR elevated (Figure 2). There was no statistical interaction between the above-normal eGFR levels and covariates regarding the incidence risk of DM (Supplementary Table S4).

In the sensitivity analysis for the population with impaired glucose tolerance, in the multivariable analysis, in comparison with the 5th decile, the 9th (102.95–109.80 mL/min/1.73 m^2^) (HR: 0.94, 95% CI (0.91–0.97), *p* < 0.001) and 10th (≥109.91 mL/min/1.73 m^2^) (HR: 0.66, 95% CI (0.62–0.69), *p* < 0.001) eGFR deciles were significantly associated with a decreased incidence of new-onset DM (Table 3). Moreover, eGFR >120 mL/min/1.73 m^2^ was associated with a reduced risk of new-onset DM (HR: 0.55, 95% CI (0.51–0.59), *p* < 0.001) (Table 3). Regardless of whether eGFR was measured using the MDRD method, the correlation between higher-than-normal eGFR levels and risk for incidence of new-onset DM remained consistently evident in the sensitivity analyses (Supplementary Table S5). In the subgroup analysis, the consistent observation of a relationship between higher-than-normal eGFR and a reduced risk of new-onset DM was noted in the age < 65 group and age ≥ 65 group (Supplementary Tables S6 and S7).

Regarding body mass index, in obese participants (body mass index ≥ 25), compared to the 5th decile, the 10th eGFR decile had a significant association with a decreased incidence of new-onset DM (HR: 0.66, 95% CI (0.64–0.69), *p* < 0.001). Furthermore, eGFR > 120 mL/min/1.73 m^2^ was related to a decreased risk of new-onset DM in obese participants (HR: 0.56, 95% CI (0.53–0.59), *p* < 0.001) (Supplementary Tables S8 and S9).

## 4. Discussion

The main findings from our investigation were that individuals with eGFR 10th decile (≥114.12 mL/min/1.73 m^2^) or those with eGFR > 120 mL/min/1.73 m^2^ had an association with decreased risk of incidence for new-onset DM, except in the ≥ 65 age groups.

A higher-than-normal eGFR is often considered as an early stage in the development of CKD in patients with DM [23]. Approximately 50% of DM patients have been observed to have renal hyperfiltration [24,25]. Renal hyperfiltration in DM patients is associated with impaired renal function, adverse cardiovascular outcomes, and increased mortality [5,26]. However, the significance of higher-than-normal eGFR in the general non-diabetic population remains unclear. Glomerular hyperfiltration, characterized by an increase in single-nephron GFR (SNGFR), is a functional and potentially reversible hemodynamic change. The extent of the increase in SNGFR is closely related to the amount of lost renal function; it is the result of activation to compensate for the total amount of lost renal function [27]. This adaptive renal hyperfiltration in surviving glomeruli is considered beneficial because it helps to minimize the reduction in total eGFR

There are several theories regarding renal hyperfiltration in the context of DM or hyperglycemia, including tubular theories, neurohormonal activation, and nephromegaly [23]. However, few theories can fully explain the mechanisms or effects of renal hyperfiltration without considering the impact of increased systemic glucose. Morphological changes may eventually damage the structural and functional integrity of the remaining glomeruli in nephrectomy cases [28]. In the case of DM, factors such as increased glucose load, concurrent tubular hypertrophy, and the upregulation of sodium–glucose cotransporter 2 (SGLT2) and sodium–hydrogen exchanger (NHE) 3 contribute to the phenomenon of renal hyperfiltration [29]. Research has shown that renal SGLT2 expression is elevated in both human cells and some animal models of Type 1 and Type 2 DM [30]. This maladaptive upregulation of SGLT2 perpetuates high blood glucose levels, thereby heightening the risks associated with DM. In non-diabetic individuals, structural changes result in increased glucose excretion. Without the SGLT2 upregulation seen in DM, inhibiting glucose reabsorption in the proximal tubule leads to greater glucose excretion in the urine, thereby lowering elevated blood glucose levels, which may explain this study’s findings. There are no reports indicating that SGLT2 upregulation accompanies impaired glucose tolerance (IGT), a pre-diabetic condition. Therefore, results similar to those in non-diabetic individuals can be expected. In this study, sensitivity analyses also showed that the IGT patient group exhibited patterns similar to those of the general patient population.

Obesity is known to be closely linked to hyperfiltration, and several mechanisms may be involved in this association. First, obesity is often associated with other renal risk factors such as hypertension and diabetes mellitus [31]. However, the independent effects of obesity are also likely significant. Renal hemodynamic factors have been suggested to play a role, as obesity and even mild overweight are associated with glomerular hyperfiltration, indicated by elevated GFR and/or filtration fraction, even when blood pressure and glucose tolerance are normal [32,33]. Nonetheless, it remains unclear whether these conditions are also associated with the general non-diabetic population. In this study, no independent association with BMI was observed. Other studies have also failed to show a clear link between BMI and renal hyperfiltration, suggesting that further research is needed [34].

In the context of health screening examinations for the general population, individuals can be informed that if their eGFR is higher than normal, such as 120 mL/min/1.73 m^2^ or above, their risk of developing diabetes may be relatively lower. This explanation also applies to individuals with prediabetes or obesity.

This study has some limitations that need to be recognized. Firstly, our results might be influenced by potential ethnic bias, potentially restricting the broader relevance of our conclusions to other demographic groups. It is important to conduct further studies across various racial and ethnic groups. Secondly, our study only looked at eGFR in a cross-sectional manner and did not consider measurements of cystatin C and HbA1c, which are important biomarkers for glucose control as these were not available in the NHIS database. Third, our study used the eGFR calculation formula from before the 2021 update, which may pose limitations to the interpretation of the results [16,35]. Fourth, there is also a chance that we may have overestimated the kidney function in individuals who had an eGFR > 90 mL/min/1.73 m^2^, which might affect the reliability of our results. Finally, despite our research being a comprehensive, nationwide longitudinal study, its retrospective design makes it challenging to determine cause-and-effect relationships.

## 5. Conclusions

Our study showed that higher-than-normal eGFR levels were associated with a lower risk of incidence for new-onset DM regardless of the presence of impaired glucose tolerance, age, or obesity. In general population, higher-than-normal eGFR may be associated with a lower risk of incidence of new-onset DM.

## Figures and Tables

**Table 1:** Baseline characteristics of participants.

Variables	Total (2,294,358)	
	N	Ratio or SD
Sex		
Male	1,158,730	50.50
Female	1,135,628	49.50
Age, years	47.34	±13.76
Body mass index (kg/m^2^)	23.58	±3.22
Waist circumference (cm)	79.58	±9.24
Household income		
Q1, lowest	624,115	27.20
Q2	810,596	35.33
Q3	572,394	24.95
Q4, highest	287,253	12.52
Smoking status		
Never	1,426,670	62.18
Former	323,126	14.08
Current	544,562	23.74
Alcohol consumption (days/week)		
None	1,204,436	52.50
1–4	1,005,378	43.82
≥5	84,544	3.68
Regular physical activity (days/week)		
None	1,399,277	61.00
1–4	218,263	9.50
≥5	676,818	29.50
Proteinuria		
Negative (−)	2,206,592	96.17
Positive (+)	87,766	3.83
Comorbidities		
Hypertension	301,828	13.16
Dyslipidemia	247,634	10.80
Heart failure	21,266	0.93
Myocardial infarction	4568	0.20
Valvular heart disease	5221	0.23
Cardiomyopathy	1401	0.06
Hyperthyroidism	23,467	1.02
Congenital heart disease	780	0.03
Charlson comorbidity index		
0	1,506,675	65.67
1	513,876	22.40
≥2	273,807	11.93
eGFR (decile), mL/min/1.73 m^2^		
1st (<68.61)	226,811	9.89
2nd (68.62–75.92)	227,435	9.91
3rd (75.93–81.78)	228,868	9.98
4th (81.80–86.69)	226,577	9.88
5th (86.76–91.13)	234,825	10.23
6th (91.15–96.33)	222,315	9.69
7th (96.40–101.03)	232,124	10.12
8th (101.04–106.38)	226,764	9.88
9th (106.46–114.02)	237,830	10.36
10th (≥114.12)	230,809	10.06
eGFR (range), mL/min/1.73 m^2^		
<30	1296	0.06
30–60	76,021	3.31
60–90	1,005,517	43.83
90–120	1,106,801	48.24
>120	104,723	4.56

Data are expressed as the mean ± standard deviation, or number (percentage). SD, standard deviation; Q, quartile; eGFR, estimated glomerular filtration rate.

**Table 2:** Association of renal function with incidence of diabetes mellitus.

HR, Hazard Ratio; CI, Confidence Interval.	Number of Participants	Number of Events	Event Rate (%) (95% CI)	Person-Years	Incidence Rate (Per 1000 Person-Years)	Adjusted HR (95% CI)	*p*-Value
eGFR (decile)							
1st (<66.24)	55,212	10,238	18.54 (18.22, 18.87)	465,420.58	21.99	1.16 (1.13, 1.19)	<0.001
2nd (66.26–73.51)	55,438	8902	16.06 (15.75, 16.36)	486,498.62	18.30	1.15 (1.12, 1.19)	<0.001
3rd (73.61–78.86)	55,506	8025	14.46 (14.17, 14.75)	493,785.82	16.25	1.14 (1.11, 1.18)	<0.001
4th (78.96–83.58)	55,874	7733	13.93 (13.64, 14.22)	492,762.15	15.69	1.09 (1.05, 1.12)	<0.001
5th (83.70–88.35)	55,781	7205	12.92 (12.64, 13.20)	498,780.04	14.45	1 (ref)	
6th (88.40–92.81)	55,180	8285	15.01 (14.72, 15.31)	488,715.31	16.95	1.20 (1.16, 1.24)	<0.001
7th (92.86–97.85)	56,860	8045	14.15 (13.86, 14.44)	507,916.24	15.84	1.18 (1.15, 1.22)	<0.001
8th (98.1–102.79)	54,239	7121	13.13 (12.85, 13.41)	487,489.01	14.61	1.15 (1.11, 1.19)	<0.001
9th (102.95–109.80)	55,205	5563	10.08 (9.83, 10.33)	503,830.77	11.04	0.94 (0.91, 0.97)	<0.001
10th (≥109.91)	55,701	3820	6.86 (6.65, 7.07)	514,763.91	7.42	0.66 (0.62, 0.69)	<0.001
eGFR (range)							
<30	1296	245	18.9 (16.77, 21.04)	8771.99	27.93	1.66 (1.47, 1.89)	<0.001
<30	407	90	22.11 (18.08, 26.14)	2569.83	35.02	1.26 (1.02, 1.55)	0.029
30–60	25,861	4873	18.84 (18.37, 19.32)	210,372.17	23.16	0.99 (0.97, 1.03)	0.867
60–90	274,358	39,866	14.53 (14.40, 14.66)	2,428,359.27	16.42	1 (ref)	
90–120	241,347	29,308	12.14 (12.01, 12.27)	2,177,657.39	13.46	0.96 (0.95, 0.98)	<0.001

CI, confidence interval; HR, hazard ratio; eGFR, estimated glomerular filtration rate.

**Table 3:** Association of renal function with incidence of diabetes mellitus in impaired glucose tolerance group.

	Number of Participants	Number of Events	Event Rate (%) (95% CI)	Person-Years	Incidence Rate (Per 1000 Person-Years)	Adjusted HR (95% CI)	*p*-Value
eGFR (decile)							
1st (<66.24)	55,212	10238	18.54 (18.22, 18.87)	465,420.58	21.99	1.16 (1.13, 1.19)	<0.001
2nd (66.26–73.51)	55,438	8902	16.06 (15.75, 16.36)	486,498.62	18.30	1.15 (1.12, 1.19)	<0.001
3rd (73.61–78.86)	55,506	8025	14.46 (14.17, 14.75)	493,785.82	16.25	1.14 (1.11, 1.18)	<0.001
4th (78.96–83.58)	55,874	7733	13.93 (13.64, 14.22)	492,762.15	15.69	1.09 (1.05, 1.12)	<0.001
5th (83.70–88.35)	55,781	7205	12.92 (12.64, 13.20)	498,780.04	14.45	1 (ref)	
6th (88.40–92.81)	55,180	8285	15.01 (14.72, 15.31)	488,715.31	16.95	1.20 (1.16, 1.24)	<0.001
7th (92.86–97.85)	56,860	8045	14.15 (13.86, 14.44)	507,916.24	15.84	1.18 (1.15, 1.22)	<0.001
8th (98.1–102.79)	54,239	7121	13.13 (12.85, 13.41)	487,489.01	14.61	1.15 (1.11, 1.19)	<0.001
9th (102.95–109.80)	55,205	5563	10.08 (9.83, 10.33)	503,830.77	11.04	0.94 (0.91, 0.97)	<0.001
10th (≥109.91)	55,701	3820	6.86 (6.65, 7.07)	514,763.91	7.42	0.66 (0.62, 0.69)	<0.001
eGFR (range)							
<30	407	90	22.11 (18.08, 26.14)	2569.83	35.02	1.26 (1.02, 1.55)	0.029
30–60	25861	4873	18.84 (18.37, 19.32)	210,372.17	23.16	0.99 (0.97, 1.03)	0.867
60–90	274,358	39,866	14.53 (14.40, 14.66)	2,428,359.27	16.42	1 (ref)	
90–120	241,347	29,308	12.14 (12.01, 12.27)	2,177,657.39	13.46	0.96 (0.95, 0.98)	<0.001
>120	13,023	800	6.14 (5.73, 6.56)	121,003.79	6.61	0.55 (0.51, 0.59)	<0.001

CI, confidence interval; HR, hazard ratio; eGFR, estimated glomerular filtration rate.

## Data Availability

The data used in this study are available in the National Health Insurance Service-National Health Screening Cohort (NHIS-HEALS) database, but restrictions apply to the public availability of these data used under license for the current study. Requests for access to the NHIS data can be made through the National Health Insurance Sharing Service homepage [http://nhiss.nhis.or.kr/bd/ab/bdaba021eng.do, accessed on 1 July 2024]. For access to the database, a completed application form, research proposal, and application for approval from the Institutional Review Board should be submitted to the inquiry committee of research support of the NHIS for review.

## Supplementary Materials

The following supporting information can be downloaded at https://www.mdpi.com/article/10.3390/jcm13175267/s1: Table S1: Baseline characteristics of participants by renal hyperfiltration (eGFR range); Table S2: Association factors of renal hyperfiltration (decile) with occurrence of diabetes mellitus; Table S3: Association factors of renal hyperfiltration (range) with occurrence of diabetes mellitus; Table S4: Statistical interaction between the demographic factors and renal hyperfiltration regarding the incidence risk of diabetes mellitus; Table S5: Association of renal function (MDRD method) with occurrence of diabetes mellitus; Table S6: Age-specific comparative analysis for association of renal hyperfiltration (decile) with incidence of diabetes mellitus; Table S7: Age-specific comparative analysis for association of renal hyperfiltration (range) with incidence of diabetes mellitus; Table S8: BMI-specific comparative analysis for association of renal hyperfiltration (decile) with diabetes mellitus; Table S9: BMI-specific comparative analysis for association of renal hyperfiltration (range) with diabetes mellitus. Figure S1: Kaplan-Meier survival curves demonstrating the relationship between estimated glomerular filtration rate (eGFR) and incidence of new-onset DM (A: deciles, B: ranges).
