# Supplementary material for: Association of Renal Hyperfiltration with Incidence of New-Onset Diabetes Mellitus: A Nationwide Cohort Study
Source: J Clin Med. 2024 Sep 5;13(17):5267. doi: 10.3390/jcm13175267 (PMC11396438; doi:10.3390/jcm13175267)
Supplement: Supplementary file 1 [file jcm-13-05267-s001.zip › Supplementary methods.pdf]

Title: **Association of higher-than-normal estimated glomerular filtration rate with incidence of diabetes mellitus: A Nationwide Cohort Study**

Running title: Association of higher-than-normal eGFR with DM

Authors

Min-Ju Kim[1*], Min Kyoung Kang[1*], Ye-Sun Hong[2*], Gwang Hyun Leem[3], Tae-Jin Song[1]

[1]Department of Neurology, Seoul Hospital, Ewha Womans University College of Medicine, Seoul, Republic of Korea

[2]Department of Physiology, Ewha Womans University College of Medicine, Seoul, Republic of Korea

[3]Department of Convergence Medicine, Seoul Hospital, Ewha Womans University College of Medicine, Seoul, Republic of Korea

*: equally contributed.

Corresponding author:

Tae-Jin Song, MD, PhD

Department of Neurology, Seoul Hospital, Ewha Womans University College of Medicine, 260, Gonghang-daero, Gangseo-gu, 07804, Seoul, Republic of Korea

Tel: +82-2-6986-1672, Fax: +82-2-6986-7000; E-mail: knstar@ewha.ac.kr

ORCID ID: 0000-0002-9937-762X

Supplementary Methods

The Chronic Kidney Disease Epidemiology Collaboration (CKD-EPI) equation was calculated as eGFR (mL/min/1.73m^2):

eGFR=144×(SCr/0.7)$^{-0.329}$× (0.993)^{age} (if female and SCr≤0.7 mg/dL),
eGFR=144×(SCr/0.7)$^{-1.209}$×(0.993)^{age} (if female and SCr>0.7 mg/dL),
eGFR=144×(SCr/0.9)$^{-0.411}$×(0.993)^{age} (if male and SCr≤0.9 mg/dL), and
eGFR=144×(SCr>0.9)$^{-1.209}$× (0.993)^{age} (if male and SCr>0.9 mg/dL) [1].

We identified hypertension according to the following criteria: 1) prescription of any antihypertensive medication with at least one claim of the diagnostic codes I10–15, 2) two or more claims of the diagnostic codes I10–15, 3) blood pressure ≥140/90 mmHg (systolic/diastolic), or 4) self-reported hypertension in the questionnaire. DM was defined as meeting one of the following criteria: 1) prescription of an antidiabetic agent with at least one claim of the diagnostic codes E11–14, 2) ≥2 claims of the diagnostic codes E11–14, 3) fasting serum glucose level≥7.0 mmol/L, or 4) self-reported DM in the questionnaire. Dyslipidemia was defined as satisfying one of the following criteria: 1) prescription of a dyslipidemia-related agent with at least one claim of the diagnostic code E78, 2) two or more claims of the diagnostic code E78, or 3) total cholesterol≥240 mg/dL. Charlson comorbidity conditions were collected using the Korean version of the Charlson comorbidity index [2-8]. Heart failure was defined as at least one claim of the diagnostic codes I50, I50.x, I11.0, I13.1, I 13.0, I13.2 [9]. Myocardial infarction failure was defined as at least one claim of the diagnostic codes I21 and I22 [10]. Valvular heart disease was defined as at least one claim of the diagnostic codes A52.0, I05, I08, I09.1, I09.8, I34, I.39, Q23.0-23.3, Z95.2-95.4 [8]. Cardiomyopathy was defined as at least one claim of the diagnostic code I42 [11]. Congenital heart disease was defined as at least one claim of the diagnostic codes: ventricular septal defect (Q21.0, Q21.00, Q21.01, Q21.08, Q21.09), atrial septal defect (Q21.1, Q21.10, Q21.11, 21.18, Q21.19), atrioventricular septal defects (Q21.2), congenital malformation of the cardiac septum (Q21.9), patent ductus arteriosus (Q25.0), pulmonary artery stenosis (Q22.1, Q25.6), coarctation of aorta (Q25.1), pulmonary venous connection (Q26.2, Q26.3, Q26.4), congenital tricuspid stenosis (Q22.4, Q22.8, Q22.9), congenital stenosis of aortic valve (Q23.0), congenital insufficiency of aortic valve (Q23.1), congenital mitral stenosis (Q23.2, Q23.3), malformation of coronary vessels (Q24.5, Q24.8, Q24.9), stenosis or malformation of aorta (Q24.4, Q25.2, Q25.3, Q25.4, Q25.8, Q25.9), Tetralogy of Fallot (Q21.3), Ebstein anomaly (Q22.5), transposition of great arteries (Q20.3, Q20.5), Eisenmenger syndrome (Q21.81, I21.81), double outlet right ventricle (Q20.1), and single ventricle (Q20.4) [12]. Hyperthyroidism was defined as at least one claim of the diagnostic code E05 [13].

Supplementary Figures

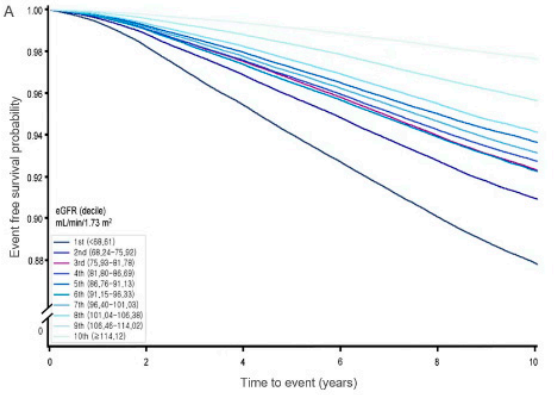

1st	226811	219456	208930	198172	187264	76403
2nd	227435	223293	216864	210031	202761	76701
3rd	228868	225678	220806	215306	209579	75705
4th	226577	223308	218418	212876	207000	71795
5th	234825	231932	227616	222645	217380	75060
6th	222315	219187	214269	209131	203651	70163
7th	232124	229497	225414	221016	216345	72339
8th	226764	224685	221548	217819	213846	69030
9th	237830	236388	233998	231154	227924	74005
10th	230809	229943	228670	227167	225462	66604

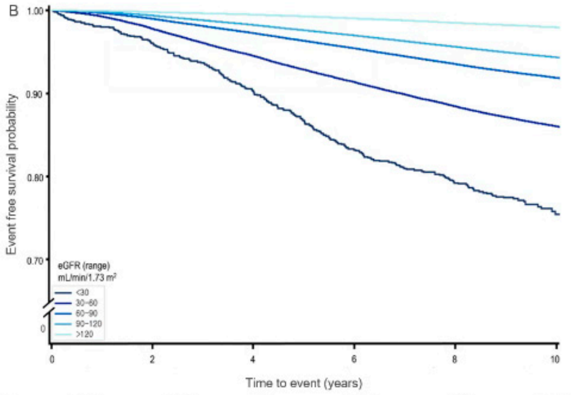

<30	1296	1116	938	786	673	215
30-60	76021	72495	67735	62977	58191	23911
60-90	1005517	989024	963899	936335	907391	331920
90-120	1106801	1096381	1080090	1061956	1042382	341935
>120	104723	104351	103871	103263	102575	29824

Supplementary Figure S1. Kaplan-Meier survival curves demonstrating the relationship between estimated glomerular filtration rate (eGFR) and incidence of new-onset DM (A: deciles, B: ranges).

References

1. Tent, H., et al., Performance of MDRD study and CKD-EPI equations for long-term follow-up of nondiabetic patients with chronic kidney disease. Nephrol Dial Transplant, 2012. 27 Suppl 3: p. iii89-95.

2. Song, T.J., J.W. Kim, and J. Kim, Oral health and changes in lipid profile: A nationwide cohort study. J Clin Periodontol, 2020. 47(12): p. 1437-1445.

3. Woo, H.G., et al., Association of Tooth Loss with New-Onset Parkinson's Disease: A Nationwide Population-Based Cohort Study. Parkinson’s Dis, 2020. 2020: p. 4760512.

4. Chang, Y., et al., Better oral hygiene is associated with lower risk of stroke. J Periodontol, 2021. 92(1): p. 87-94.

5. Lee, K., et al., Oral health and gastrointestinal cancer: A nationwide cohort study. J Clin Periodontol, 2020. 47(7): p. 796-808.

6. Kim, J., et al., Association between oral health and cardiovascular outcomes in patients with hypertension: a nationwide cohort study. J Hypertens, 2022. 40(2): p. 374-381.

7. Song, T.J., et al., Oral health and longitudinal changes in fasting glucose levels: A nationwide cohort study. PLoS One, 2021. 16(6): p. e0253769.

8. Kim, K.H. Comorbidity Adjustment in Health Insurance Claim Database. Health Policy and Management. 2016;26(1):71-78

9. Park, J. J. et al. Heart Failure Statistics in Korea, 2020: A Report from the Korean Society of Heart Failure. Int J Heart Fail 3, 224-236 (2021).

10. Choi, E. K. Cardiovascular Research Using the Korean National Health Information Database. Korean Circ J 50, 754-772 (2020).

11. Lee, J. H., Lim, N. K., Cho, M. C. & Park, H. Y. Epidemiology of Heart Failure in Korea: Present and Future. Korean Circ J 46, 658-664 (2016).

12. Jang, S. Y. et al. Prevalence and mortality of congenital heart disease in Korean adults. Medicine (Baltimore) 97, e11348 (2018).

13. Lee, S. R. et al. Improved prognosis with integrated care management including early rhythm control and healthy lifestyle modification in patients with concurrent atrial fibrillation and diabetes mellitus: a nationwide cohort study. Cardiovasc Diabetol 22, 18 (2023).
