# Supplementary material for: Association of Renal Hyperfiltration with Incidence of New-Onset Diabetes Mellitus: A Nationwide Cohort Study
Source: J Clin Med. 2024 Sep 5;13(17):5267. doi: 10.3390/jcm13175267 (PMC11396438; doi:10.3390/jcm13175267)
Supplement: Supplementary file 1 [file jcm-13-05267-s001.zip › Supplementary tables.pdf]

Supplementary Table S1. Baseline characteristics of participants by renal hyperfiltration (eGFR range)

Variables	Total (2,294,358)		< 30		30-60		60-90		90-120		>120		
	N	% or SD	N	% or SD	N	% or SD	N	% or SD	N	% or SD	N	% or SD	*p*
Sex													<.001
Male	1,158,730	50.50	621	47.92	30,868	40.61	524,408	52.15	564,409	50.99	38,424	36.69	
Female	1,135,628	49.50	675	52.08	45,153	59.39	481,109	47.85	542,392	49.01	66,299	63.31	
Age, years	47.34	± 13.76	66.73	± 15.18	65.68	± 11.95	51.66	± 13.24	43.74	± 11.68	30.33	± 7.92	<.001
Body mass index (kg/m^2)	23.58	± 3.22	23.69	± 3.35	24.15	± 3.18	23.84	± 3.10	23.43	± 3.25	22.25	± 3.56	<.001
Waist circumference (cm)	79.58	± 9.24	82.37	± 9.72	82.33	± 8.76	80.52	± 9.08	78.98	± 9.13	74.93	± 10.16	<.001
Household income													<.001
Q1, lowest	624,115	27.20	311	24.00	20,167	26.53	256,506	25.51	306,689	27.71	40,442	38.62	
Q2	810,596	35.33	394	30.40	21,795	28.67	327,777	32.60	414,191	37.42	46,439	44.34	
Q3	572,394	24.95	349	26.93	20,295	26.70	269,322	26.78	268,093	24.22	14,335	13.69	
Q4, highest	287,253	12.52	242	18.67	13,764	18.10	151,912	15.11	117,828	10.65	3,507	3.35	
Smoking status													<.001
Never	1,426,670	62.18	917	70.76	55,675	73.24	625,324	62.19	672,194	60.73	72,560	69.29	
Former	323,126	14.08	209	16.13	11,484	15.10	162,142	16.13	141,540	12.79	7,751	7.40	
Current	544,562	23.74	170	13.11	8,862	11.66	218,051	21.68	293,067	26.48	24,412	23.31	
Alcohol consumption (days/week)													<.001
None	1,204,436	52.50	1018	78.55	56,706	74.59	558,020	55.49	541,882	48.96	46,810	44.70	
1–4	1,005,378	43.82	251	19.37	16,516	21.73	407,402	40.52	525,313	47.46	55,896	53.38	
≥5	84,544	3.68	27	2.08	2,799	3.68	40,095	3.99	39,606	3.58	2,017	1.92	
Regular physical activity (days/week)													<.001
None	1,399,277	60.99	993	76.62	52,738	69.37	603,567	60.03	674,032	60.90	67,947	64.88	
1–4	218,263	9.51	66	5.09	6,521	8.58	103,113	10.25	101,368	9.16	7,195	6.87	
≥5	676,818	29.50	237	18.29	16,762	22.05	298,837	29.72	331,401	29.94	29,581	28.25	
Proteinuria													<.001
Negative (-)	2,206,592	96.17	776	59.88	69,777	91.79	966,244	96.09	1,068,926	96.58	100,869	96.32	
Positive (+)	87,766	3.83	520	40.12	6,244	8.21	39,273	3.91	37,875	3.42	3,854	3.68	
Comorbidities													
Hypertension													<.001
No	1,992,530	86.84	908	70.06	57,018	75.00	852,547	84.79	982,831	88.80	99,226	94.75	
Yes	301,828	13.16	388	29.94	19,003	25.00	152,970	15.21	123,970	11.20	5,497	5.25	
Dyslipidemia													<.001
No	2,046,724	89.20	1,140	87.96	63,804	83.93	877,792	87.30	1,004,185	90.73	99,803	95.30	
Yes	247,634	10.80	156	12.04	12,217	16.07	127,725	12.70	102,616	9.27	4,920	4.70	
Heart failure													<.001
No	2,273,092	99.07	1,132	87.35	72,164	94.93	993,644	98.82	1,101,557	99.53	104,595	99.88	
Yes	21,266	0.93	164	12.65	3,857	5.07	11873	1.18	5,244	0.47	128	0.12	

Myocardial Infarction													<.001
No	2,289,790	99.80	1,264	97.53	75,349	99.12	1,002,922	99.74	1,105,570	99.89	104,685	99.96	
Yes	4,568	0.20	32	2.47	672	0.88	2595	0.26	1,231	0.11	38	0.04	
Valvular heart disease													<.001
No	2,289,137	99.77	1,268	97.84	75,225	98.95	1,002,696	99.72	1,105,286	99.86	104,662	99.94	
Yes	5,221	0.23	28	2.16	796	1.05	2821	0.28	1,515	0.14	61	0.06	
Cardiomyopathy													<.001
No	2,292,957	99.94	1,279	98.69	75,770	99.67	1,004,766	99.93	1,106,434	99.97	104,708	99.99	
Yes	1,401	0.06	17	1.31	251	0.33	751	0.07	367	0.03	15	0.01	
Hyperthyroidism													<.001
No	2,270,891	98.98	1,277	98.53	75,061	98.74	995,522	99.01	1,095,377	98.97	103,654	98.98	
Yes	23,467	1.02	19	1.47	960	1.26	9995	0.99	11,424	1.03	1069	1.02	
Congenital heart disease													<.001
No	2,293,578	99.97	1,294	99.85	75,974	99.94	1,005,167	99.97	1,106,458	99.97	104,685	99.96	
Yes	780	0.03	2	0.15	47	0.06	350	0.03	343	0.03	38	0.04	
Charlson comorbidity index													<.001
0	1,506,675	65.67	289	22.3	31,076	40.88	623,087	61.97	769,499	69.52	82,724	78.99	
1	513,876	22.40	253	19.52	21,230	27.93	239,716	23.84	234,895	21.22	17,782	16.98	
≥2	273,807	11.93	754	31.19	23,715	31.19	142,714	14.19	102,407	9.25	4,217	4.03	

Data is presented as the mean ± standard deviation, or as a number (percentage).

SD: standard deviation, Q: quartile. eGFR: Estimated Glomerular Filtration Rate.

Supplementary Table S2. Association factors of renal hyperfiltration (decile) with occurrence of diabetes mellitus

Variable	Crude HR (95% CI)	p-value	Adjusted HR (95% CI)	p-value
Sex				
Male	1 (ref)		1 (ref)	
Female	1.01 (1.00, 1.02)	0.0756	1.08 (1.06, 1.09)	<.001
Age, years				
< 65	1 (ref)		1 (ref)	
≥65	2.72 (2.69, 2.75)	<.001	1.64 (1.62, 1.67)	<.001
Household income				
Q1, lowest	1 (ref)		1 (ref)	
Q2	0.89 (0.88, 0.91)	<.001	0.91 (0.90, 0.93)	<.001
Q3	1.02 (1.00, 1.03)	0.024	0.92 (0.91, 0.93)	<.001
Q4, highest	1.18 (1.16, 1.2)	<.001	0.98 (0.97, 1.00)	0.025
Smoking status				
Never	1 (ref)		1 (ref)	
Former	1.15 (1.13, 1.17)	<.001	1.15 (1.13, 1.17)	<.001
Current	0.93 (0.92, 0.94)	<.001	1.20 (1.18, 1.22)	<.001
Alcohol consumption (days/week)				
None	1 (ref)		1 (ref)	
1-4	0.70 (0.69, 0.71)	<.001	0.83 (0.82, 0.84)	<.001
≥ 5	1.23 (1.20, 1.26)	<.001	1.12 (1.09, 1.15)	<.001
Regular physical activity (days/week)				
None	1 (ref)		1 (ref)	
1-4	0.97 (0.96, 0.99)	0.003	1.01 (1.00, 1.03)	0.136
≥ 5	0.88 (0.87, 0.89)	<.001	0.97 (0.96, 0.98)	<.001
Body mass index (kg/m^2)				
< 25	1 (ref)		1 (ref)	
≥ 25	2.34 (2.32, 2.37)	<.001	1.88 (1.86, 1.90)	<.001
Waist circumference (cm)	1.01 (1.01, 1.01)	<.001	1.01 (1.01, 1.01)	<.001
Proteinuria				
Negative (-)	1 (ref)		1 (ref)	
Positive (+)	1.55 (1.51, 1.58)	<.001	1.32 (1.29, 1.34)	<.001
Comorbidities				
Hypertension	2.25 (2.22, 2.27)	<.001	1.54 (1.52, 1.56)	<.001

Dyslipidemia	1.69 (1.66, 1.71)	<.001	1.37 (1.35, 1.39)	<.001
Heart failure	2.88 (2.79, 2.98)	<.001	1.13 (1.10, 1.18)	<.001
Myocardial Infarction	2.90 (2.7, 3.11)	<.001	1.37 (1.27, 1.47)	<.001
Valvular heart disease	1.93 (1.78, 2.1)	<.001	1.05 (0.97, 1.14)	0.208
Cardiomyopathy	2.54 (2.22, 2.92)	<.001	1.18 (1.03, 1.36)	0.019
Hyperthyroidism	1.54 (1.48, 1.61)	<.001	1.30 (1.25, 1.36)	<.001
Congenital heart disease	1.30 (1.02, 1.66)	0.040	0.97 (0.76, 1.25)	0.835
Charlson comorbidity index				
0	1 (ref)		1 (ref)	
1	1.75 (1.73, 1.77)	<.001	1.50 (1.48, 1.52)	<.001
≥ 2	2.98 (2.95, 3.02)	<.001	2.09 (2.07, 2.12)	<.001
eGFR, decile				
1st (< 68.61)	1.99 (1.95, 2.04)	<.001	1.34 (1.31, 1.37)	<.001
2nd (68.62-75.92)	1.45 (1.42, 1.48)	<.001	1.25 (1.23, 1.28)	<.001
3rd (75.93-81.78)	1.22 (1.19, 1.25)	<.001	1.22 (1.19, 1.25)	<.001
4th (81.80-86.69)	1.15 (1.12, 1.17)	<.001	1.13 (1.11, 1.16)	<.001
5th (86.76-91.13)	1 (ref)		1 (ref)	
6th (91.15-96.33)	1.22 (1.20, 1.25)	<.001	1.24 (1.21, 1.27)	<.001
7th (96.40-101.03)	1.08 (1.06, 1.11)	<.001	1.20 (1.17, 1.23)	<.001
8th (101.04-106.38)	0.92 (0.90, 0.94)	<.001	1.11 (1.09, 1.14)	<.001
9th (106.46-114.02)	0.67 (0.65, 0.69)	<.001	0.84 (0.82, 0.86)	<.001
10th (≥ 114.12)	0.36 (0.35, 0.37)	<.001	0.52 (0.50, 0.54)	<.001

The multivariable model was adjusted for sex, age, body mass index, waist circumference, income levels, smoking, alcohol consumption, regular physical activity, proteinuria, hypertension, dyslipidemia, heart failure, myocardial infarction, valvular heart disease, cardiomyopathy, hyperthyroidism, congenital heart disease, and Charlson comorbidity index.

HR, hazard ratio; CI, confidence interval; Q, quartile; eGFR, Estimated Glomerular Filtration Rate.

Supplementary Table S3. Association factors of renal hyperfiltration (range) with occurrence of diabetes mellitus

Variable	Crude HR (95% CI)	p-value	Adjusted HR (95% CI)	p-value
Sex				
Male	1 (ref)		1 (ref)	
Female	1.01 (1.00, 1.02)	0.075	1.07 (1.06, 1.09)	<.001
Age, years				
< 65	1 (ref)		1 (ref)	
≥65	2.72 (2.69, 2.75)	<.001	1.67 (1.65, 1.69)	<.001
Household income				
Q1, lowest	1 (ref)		1 (ref)	
Q2	0.89 (0.88, 0.91)	<.001	0.91 (0.90, 0.92)	<.001
Q3	1.02 (1.00, 1.03)	0.024	0.92 (0.91, 0.94)	<.001
Q4, highest	1.18 (1.16, 1.20)	<.001	0.99 (0.98, 1.01)	0.441
Smoking status				
Never	1 (ref)		1 (ref)	
Former	1.15 (1.13, 1.17)	<.001	1.16 (1.14, 1.18)	<.001
Current	0.93 (0.92, 0.94)	<.001	1.19 (1.17, 1.21)	<.001
Alcohol consumption (days/week)				
None	1 (ref)		1 (ref)	
1-4	0.70 (0.69, 0.71)	<.001	0.82 (0.81, 0.83)	<.001
≥ 5	1.23 (1.2, 1.26)	<.001	1.12 (1.10, 1.15)	<.001
Regular physical activity (days/week)				
None	1 (ref)		1 (ref)	
1-4	0.97 (0.96, 0.99)	0.003	1.02 (1, 1.04)	0.029
≥ 5	0.88 (0.87, 0.89)	<.001	0.97 (0.96, 0.98)	<.001
Body mass index (kg/m2)				
< 25	1 (ref)		1 (ref)	
≥ 25	2.34 (2.32, 2.37)	<.001	1.89 (1.87, 1.91)	<.001
Waist circumference (cm)	1.01 (1.01, 1.01)	<.001	1.01 (1.01, 1.01)	<.001
Proteinuria				
Negative (-)	1 (ref)		1 (ref)	
Positive (+)	1.55 (1.51, 1.58)	<.001	1.31 (1.28, 1.34)	<.001
Comorbidities				

Hypertension	2.25 (2.22, 2.27)	<.0001	1.56 (1.54, 1.58)	<.001
Dyslipidemia	1.69 (1.66, 1.71)	<.0001	1.39 (1.37, 1.41)	<.001
Heart failure	2.88 (2.79, 2.98)	<.0001	1.14 (1.10, 1.18)	<.001
Myocardial Infarction	2.90 (2.7, 3.11)	<.0001	1.38 (1.28, 1.48)	<.001
Valvular heart disease	1.93 (1.78, 2.1)	<.0001	1.06 (0.98, 1.16)	0.136
Cardiomyopathy	2.54 (2.22, 2.92)	<.0001	1.18 (1.03, 1.36)	0.017
Hyperthyroidism	1.54 (1.48, 1.61)	<.0001	1.30 (1.25, 1.36)	<.001
Congenital heart disease	1.3 (1.02, 1.66)	0.0357	0.96 (0.75, 1.23)	0.7307
Charlson comorbidity index				
0	1 (ref)		1 (ref)	
1	1.75 (1.73, 1.77)	<.001	1.52 (1.51, 1.54)	<.001
≥ 2	2.98 (2.95, 3.02)	<.001	2.14 (2.11, 2.17)	<.001
eGFR, range				
<30	3.42 (3.02, 3.88)	<.001	1.66 (1.47, 1.89)	<.001
30-60	1.80 (1.76, 1.84)	<.001	1.10 (1.07, 1.12)	<.001
60-90	1 (ref)		1 (ref)	
90-120	0.68 (0.68, 0.69)	<.001	0.88 (0.87, 0.89)	<.001
>120	0.24 (0.23, 0.25)	<.001	0.40 (0.39, 0.42)	<.001

The multivariable model was adjusted for sex, age, body mass index, waist circumference, income levels, smoking, alcohol consumption, regular physical activity, proteinuria, hypertension, dyslipidemia, heart failure, myocardial infarction, valvular heart disease, cardiomyopathy, hyperthyroidism, congenital heart disease, and Charlson comorbidity index.

HR, hazard ratio; CI, confidence interval; Q, quartile; eGFR, Estimated Glomerular Filtration Rate.

Supplementary Table S4. Statistical interaction between the demographic factors and renal hyperfiltration regarding the incidence risk of diabetes mellitus.

Variable	Adjusted HR (95% CI)	p for interaction
Sex		
Male	0.96 (0.90, 1.02)	0.860
Female	0.97 (0.89, 1.04)	
Age, years		
< 65	0.94 (0.86, 1.03)	0.127
≥65	0.98 (0.92, 1.05)	
Household income		
Q1~Q2, low	1.03 (1.00, 1.06)	0.162
Q3~Q4, high	0.97 (0.92, 1.02)	
Smoking status		
Never or Former	0.82 (0.77, 0.88)	0.085
Current	0.97 (0.92, 1.02)	
Alcohol consumption (days/week)		
None	0.92 (0.83, 1.03)	0.579
≥1-4	0.96 (0.89, 1.02)	
Regular physical activity (days/week)		
None	1.03 (1.01, 1.06)	0.059
≥1	0.97 (0.96, 0.99)	
Body mass index (kg/m^2)		
< 25	0.94 (0.86, 1.02)	0.658
≥ 25	1.03 (0.92, 1.14)	
Waist circumference (cm)	0.96 (0.85, 1.07)	0.492
Proteinuria		
Negative (-)	0.95 (0.87, 1.02)	0.063
Positive (+)	1.01 (0.92, 1.09)	

HR: hazard ratio, CI: confidence interval.

Supplementary Table S5. Association of renal function (MDRD method) with occurrence of diabetes mellitus

	Adjusted HR (95% CI)	p-value
eGFR (decile), mL/min/1.73 m^2		
1st (<63.44)	1.44 (1.41, 1.48)	<.001
2nd (63.47-69.33)	1.38 (1.35, 1.42)	<.001
3rd (69.35-74.09)	1.30 (1.27, 1.33)	<.001
4th (74.14-77.75)	1.37 (1.34, 1.41)	<.001
5th (77.87-82.25)	1 (reference)	
6th (82.26-86.67)	1.34 (1.31, 1.37)	<.001
7th (86.70-91.25)	1.28 (1.25, 1.31)	<.001
8th (91.33-99.08)	0.95 (0.93, 0.98)	<.001
9th (99.12-109.63)	0.85 (0.81, 0.89)	<.001
10th (>109.64)	0.53 (0.50, 0.56)	<.001
eGFR(range), mL/min/1.73 m^2		
< 30	1.74 (1.54, 1.96)	<.001
30-60	1.12 (1.11, 1.15)	<.001
60-90	1 (reference)	
90-120	0.86 (0.85, 0.87)	<.001
>120	0.56 (0.49, 0.63)	<.001

The multivariable model was adjusted for sex, age, body mass index, waist circumference, income levels, smoking, alcohol consumption, regular physical activity, proteinuria, hypertension, dyslipidemia, heart failure, myocardial infarction, valvular heart disease, cardiomyopathy, hyperthyroidism, congenital heart disease, and Charlson comorbidity index.

HR, hazard ratio; CI, confidence interval; Q, quartile; eGFR, Estimated Glomerular Filtration

Supplementary Table S6. Age-specific comparative analysis for association of renal hyperfiltration (decile) with incidence of diabetes mellitus.

Variable	Population (N=2,294,362)		Age Group < 65y (n = 2,016,940)		Age Group ≥ 65y (n = 277,422)	
	HR CI (95%)	*p*-value	HR CI (95%)	*p*-value	HR CI (95%)	*p*-value
Sex						
Male	1 (ref)		1 (ref)		1 (ref)	
Female	1.08 (1.06, 1.09)	<.001	1.13 (1.12, 1.15)	<.001	1.03 (1, 1.06)	0.032
Household income						
Q1, lowest	1 (ref)		1 (ref)		1 (ref)	
Q2	0.91 (0.90, 0.93)	<.001	0.91 (0.90, 0.93)	<.001	0.95 (0.93, 0.98)	<.001
Q3	0.92 (0.91, 0.93)	<.001	0.93 (0.92, 0.95)	<.001	0.91 (0.89, 0.94)	<.001
Q4, highest	0.98 (0.97, 1.00)	0.025	1.01 (1.00, 1.03)	0.158	0.95 (0.92, 0.98)	<.001
Smoking status						
Never	1 (ref)		1 (ref)		1 (ref)	
Former	1.15 (1.13, 1.17)	<.001	1.16 (1.14, 1.18)	<.001	1.09 (1.05, 1.12)	<.001
Current	1.20 (1.18, 1.22)	<.001	1.23 (1.21, 1.25)	<.001	1.18 (1.14, 1.23)	<.001
Alcohol consumption (days/week)						
None	1 (ref)		1 (ref)		1 (ref)	
1-4	0.83 (0.82, 0.84)	<.001	0.81 (0.80, 0.83)	<.001	1.01 (0.98, 1.04)	0.525
≥ 5	1.12 (1.09, 1.15)	<.001	1.14 (1.11, 1.18)	<.001	1.00 (0.96, 1.05)	0.930
Regular physical activity (days/week)						
None	1 (ref)		1 (ref)		1 (ref)	
1-4	1.01 (1.00, 1.03)	0.136	1.01 (0.99, 1.03)	0.517	1.01 (0.97, 1.05)	0.653
≥ 5	0.97 (0.96, 0.98)	<.001	0.96 (0.95, 0.97)	<.001	1.02 (1, 1.05)	0.078
Body mass index (kg/m^2)						
< 25	1 (ref)		1 (ref)		1 (ref)	
≥ 25	1.88 (1.86, 1.90)	<.001	2.03 (2.00, 2.05)	<.001	1.47 (1.44, 1.5)	<.001
Waist circumference (cm)	1.01 (1.01, 1.01)	<.001	1.01 (1.01, 1.01)	<.001	1.01 (1.01, 1.01)	<.001
Proteinuria						
Negative (-)	1 (ref)		1 (ref)		1 (ref)	
Positive (+)	1.32 (1.29, 1.34)	<.001	1.35 (1.32, 1.39)	<.001	1.21 (1.16, 1.26)	<.001
Comorbidities						
Hypertension	1.54 (1.52, 1.56)	<.001	1.73 (1.71, 1.76)	<.001	1.15 (1.13, 1.18)	<.01
Dyslipidemia	1.37 (1.35, 1.39)	<.001	1.43 (1.41, 1.46)	<.001	1.13 (1.1, 1.17)	<.001
Heart failure	1.13 (1.10, 1.18)	<.001	1.37 (1.31, 1.45)	<.001	1.07 (1.02, 1.12)	0.009
Myocardial Infarction	1.37 (1.27, 1.47)	<.001	1.52 (1.38, 1.67)	<.001	1.23 (1.11, 1.37)	0.001
Valvular heart disease	1.05 (0.97, 1.14)	0.208	1.08 (0.96, 1.21)	0.211	1.02 (0.91, 1.15)	0.681
Cardiomyopathy	1.18 (1.03, 1.36)	0.019	1.29 (1.06, 1.57)	0.010	1.10 (0.9, 1.34)	0.364
Hyperthyroidism	1.30 (1.25, 1.36)	<.001	1.33 (1.27, 1.40)	<.001	1.16 (1.06, 1.27)	<.001
Congenital heart disease	0.97 (0.76, 1.25)	0.835	1.06 (0.80, 1.41)	0.695	0.82 (0.51, 1.32)	0.411
Charlson comorbidity						

index						
0	1 (ref)		1 (ref)		1 (ref)	
1	1.50 (1.48, 1.52)	<.001	1.54 (1.52, 1.56)	<.001	1.24 (1.21, 1.28)	<.001
≥ 2	2.09 (2.07, 2.12)	<.001	2.28 (2.24, 2.32)	<.001	1.57 (1.54, 1.61)	<.001
eGFR, decile						
1st	1.34 (1.31, 1.37)	<.001	1.44 (1.40, 1.47)	<.001	1.02 (0.97, 1.06)	0.497
2nd	1.25 (1.23, 1.28)	<.001	1.16 (1.13, 1.19)	<.001	0.97 (0.93, 1.01)	0.155
3rd	1.22 (1.19, 1.25)	<.001	1.26 (1.23, 1.29)	<.001	1.02 (0.97, 1.06)	0.492
4th	1.13 (1.11, 1.16)	<.001	0.98 (0.95, 1.00)	0.084	0.89 (0.85, 0.93)	<.001
5th	1 (ref)		1 (ref)		1 (ref)	
6th	1.24 (1.21, 1.27)	<.001	1.31 (1.28, 1.34)	<.001	0.95 (0.91, 0.99)	0.025
7th	1.20 (1.17, 1.23)	<.001	1.13 (1.10, 1.16)	<.001	0.93 (0.89, 0.98)	0.002
8th	1.11 (1.09, 1.14)	<.001	1.07 (1.05, 1.10)	<.001	0.97 (0.93, 1.02)	0.240
9th	0.84 (0.82, 0.86)	<.001	0.78 (0.76, 0.81)	<.001	0.95 (0.91, 1.00)	0.054
10th	0.52 (0.50, 0.54)	<.001	0.52 (0.50, 0.54)	<.001	0.93 (0.90, 0.96)	0.023

The multivariable model was adjusted for sex, body mass index, waist circumference, income levels, smoking, alcohol consumption, regular physical activity, proteinuria, hypertension, dyslipidemia, heart failure, myocardial infarction, valvular heart disease, cardiomyopathy, hyperthyroidism, congenital heart disease, and Charlson comorbidity index.

HR, hazard ratio; CI, confidence interval; Q, quartile; eGFR, Estimated Glomerular Filtration Rate.

Supplementary Table S7. Age-specific comparative analysis for association of renal hyperfiltration (range) with incidence of diabetes mellitus.

Variable	Population (N=2,294,362)		Age Group < 65y (n = 2,016,940)		Age Group ≥ 65y (n = 277,422)	
	HR CI (95%)	*p*-value	HR CI (95%)	*p*-value	HR CI (95%)	*p*-value
Sex						
Male	1 (ref)		1 (ref)		1 (ref)	
Female	1.07 (1.06, 1.09)	<.001	1.13 (1.11, 1.15)	<.001	1.05 (1.02, 1.08)	0.002
Household income						
Q1, lowest	1 (ref)		1 (ref)		1 (ref)	
Q2	0.91 (0.90, 0.92)	<.001	0.91 (0.90, 0.92)	<.001	0.95 (0.93, 0.98)	0.001
Q3	0.92 (0.91, 0.94)	<.001	0.94 (0.92, 0.95)	<.001	0.92 (0.89, 0.94)	<.001
Q4, highest	0.99 (0.98, 1.01)	0.441	1.03 (1.01, 1.05)	0.001	0.95 (0.92, 0.98)	<.001
Smoking status						
Never	1 (ref)		1 (ref)		1 (ref)	
Former	1.16 (1.14, 1.18)	<.001	1.17 (1.15, 1.19)	<.001	1.08 (1.05, 1.12)	<.001
Current	1.19 (1.17, 1.21)	<.001	1.22 (1.19, 1.24)	<.001	1.18 (1.14, 1.23)	<.001
Alcohol consumption (days/week)						
None	1 (ref)		1 (ref)		1 (ref)	
1-4	0.82 (0.81, 0.83)	<.001	0.80 (0.78, 0.81)	<.001	1.01 (0.98, 1.04)	0.532
≥ 5	1.12 (1.10, 1.15)	<.001	1.15 (1.11, 1.18)	<.001	1.00 (0.96, 1.05)	0.884
Regular physical activity (days/week)						
None	1 (ref)		1 (ref)		1 (ref)	
1-4	1.02 (1.00, 1.04)	0.029	1.01 (0.99, 1.03)	0.251	1.01 (0.97, 1.05)	0.601
≥ 5	0.97 (0.96, 0.98)	<.001	0.96 (0.94, 0.97)	<.001	1.02 (1.00, 1.05)	0.072
Body mass index (kg/m^2)						
< 25	1 (ref)		1 (ref)		1 (ref)	
≥ 25	1.89 (1.87, 1.91)	<.001	2.05 (2.02, 2.07)	<.001	1.47 (1.44, 1.50)	<.001
Waist circumference (cm)	1.01 (1.01, 1.01)	<.001	1.01 (1.01, 1.01)	<.001	1.01 (1.01, 1.01)	<.001
Proteinuria						
Negative (-)	1 (ref)		1 (ref)		1 (ref)	
Positive (+)	1.31 (1.28, 1.34)	<.001	1.34 (1.31, 1.38)	<.001	1.20 (1.15, 1.26)	<.001
Comorbidities						
Hypertension	1.56 (1.54, 1.58)	<.001	1.76 (1.74, 1.79)	<.001	1.15 (1.13, 1.18)	<.001
Dyslipidemia	1.39 (1.37, 1.41)	<.001	1.46 (1.44, 1.49)	<.001	1.13 (1.10, 1.17)	<.001
Heart failure	1.14 (1.10, 1.18)	<.001	1.41 (1.34, 1.48)	<.001	1.06 (1.01, 1.12)	0.014
Myocardial Infarction	1.38 (1.28, 1.48)	<.001	1.54 (1.39, 1.69)	<.001	1.23 (1.11, 1.37)	<.001
Valvular heart disease	1.06 (0.98, 1.16)	0.136	1.1 (0.97, 1.23)	0.129	1.02 (0.91, 1.15)	0.695
Cardiomyopathy	1.18 (1.03, 1.36)	0.017	1.29 (1.06, 1.57)	0.010	1.09 (0.89, 1.33)	0.391
Hyperthyroidism	1.3 (1.25, 1.36)	<.001	1.33 (1.27, 1.39)	<.001	1.16 (1.07, 1.27)	<.001
Congenital heart disease	0.96	0.731	1.03	0.8437	0.82	0.403

	(0.75, 1.23)		(0.77, 1.37)		(0.51, 1.31)	
Charlson comorbidity index						
0	1 (ref)		1 (ref)		1 (ref)	
1	1.52 (1.51, 1.54)	<.001	1.56 (1.54, 1.59)	<.001	1.24 (1.21, 1.28)	<.001
≥ 2	2.14 (2.11, 2.17)	<.001	2.36 (2.32, 2.40)	<.001	1.57 (1.53, 1.61)	<.001
eGFR, range						
<30	1.66 (1.47, 1.89)	<.001	2.07 (1.72, 2.49)	<.001	1.52 (1.28, 1.80)	<.001
30-60	1.10 (1.07, 1.12)	<.001	1.31 (1.27, 1.36)	<.001	1.05 (1.02, 1.08)	0.001
60-90	1 (ref)		1 (ref)		1 (ref)	
90-120	0.88 (0.87, 0.89)	<.001	0.87 (0.86, 0.88)	<.001	1.03 (1, 1.05)	0.074
>120	0.40 (0.39, 0.42)	<.001	0.42 (0.40, 0.43)	<.001	0.90 (0.83, 0.97)	0.027

The multivariable model was adjusted for sex, body mass index, waist circumference, income levels, smoking, alcohol consumption, regular physical activity, proteinuria, hypertension, dyslipidemia, heart failure, myocardial infarction, valvular heart disease, cardiomyopathy, hyperthyroidism, congenital heart disease, and Charlson comorbidity index.

HR, hazard ratio; CI, confidence interval; Q, quartile; eGFR, Estimated Glomerular Filtration Rate.

Supplementary Table S8. BMI-specific comparative analysis for association of renal hyperfiltration (decile) with diabetes mellitus

Variable	Population (N=2,294,362)		BMI Group < 25 (n=1,579,452)		BMI Group ≥ 25 (n=714,906)	
	HR CI (95%)	*p*-value	HR CI (95%)	*p*-value	HR CI (95%)	*p*-value
Sex						
Male	1 (ref)		1 (ref)		1 (ref)	
Female	1.08 (1.06, 1.09)	<.001	1.03 (1.01, 1.05)	0.016	1.20 (1.18, 1.23)	<.001
Age, years						
< 65	1 (ref)		1 (ref)		1 (ref)	
≥65	1.64 (1.62, 1.67)	<.001	1.84 (1.81, 1.88)	<.001	1.39 (1.37, 1.42)	<.001
Household income						
Q1, lowest	1 (ref)		1 (ref)		1 (ref)	
Q2	0.91 (0.90, 0.93)	<.001	0.91 (0.9, 0.93)	<.001	0.92 (0.90, 0.93)	<.001
Q3	0.92 (0.91, 0.93)	<.001	0.94 (0.92, 0.96)	<.001	0.90 (0.89, 0.92)	<.001
Q4, highest	0.98 (0.97, 0.99)	0.025	1.00 (0.98, 1.03)	0.92	0.97 (0.95, 0.99)	0.006
Smoking status						
Never	1 (ref)		1 (ref)		1 (ref)	
Former	1.15 (1.13, 1.17)	<.001	1.17 (1.14, 1.2)	<.001	1.13 (1.10, 1.16)	<.001
Current	1.20 (1.18, 1.22)	<.001	1.21 (1.18, 1.24)	<.001	1.17 (1.15, 1.20)	<.001
Alcohol consumption (days/week)						
None	1 (ref)		1 (ref)		1 (ref)	
1-4	0.83 (0.82, 0.84)	<.001	0.82 (0.81, 0.84)	<.001	0.85 (0.84, 0.87)	<.001
≥ 5	1.12 (1.09, 1.15)	<.001	1.18 (1.14, 1.22)	<.001	1.06 (1.02, 1.10)	0.002
Regular physical activity (days/week)						
None	1 (ref)		1 (ref)		1 (ref)	
1-4	1.01 (1, 1.03)	0.136	1.06 (1.03, 1.08)	<.001	0.98 (0.95, 1.00)	0.050
≥ 5	0.97 (0.96, 0.98)	<.001	0.99 (0.98, 1.01)	0.502	0.95 (0.93, 0.96)	<.001
Waist circumference (cm)	1.01 (1.01, 1.01)	<.001	1.01 (1.01, 1.01)	<.001	1.01 (1.01, 1.01)	<.001
Proteinuria						
Negative (-)	1 (ref)		1 (ref)		1 (ref)	
Positive (+)	1.32 (1.29, 1.34)	<.001	1.30 (1.26, 1.34)	<.001	1.34 (1.30, 1.38)	<.001
Comorbidities						
Hypertension	1.54 (1.52, 1.56)	<.001	1.63 (1.60, 1.66)	<.001	1.46 (1.44, 1.49)	<.001
Dyslipidemia	1.37 (1.35, 1.39)	<.001	1.57 (1.53, 1.60)	<.001	1.21 (1.19, 1.23)	<.001
Heart failure	1.13 (1.10, 1.18)	<.001	1.13 (1.07, 1.19)	<.001	1.17 (1.12, 1.23)	<.001
Myocardial Infarction	1.37 (1.27, 1.47)	<.001	1.42 (1.28, 1.58)	<.001	1.34 (1.22, 1.48)	<.001
Valvular heart disease	1.05 (0.97, 1.14)	0.208	1.13 (1.01, 1.25)	0.029	0.94 (0.82, 1.06)	0.314
Cardiomyopathy	1.18 (1.03, 1.36)	0.019	1.24 (1.02, 1.52)	0.036	1.11(0.95, 1.39)	0.155
Hyperthyroidism	1.30 (1.25, 1.36)	<.001	1.40 (1.32, 1.47)	<.001	1.20 (1.12, 1.27)	<.001
Congenital heart disease	0.97 (0.76, 1.25)	0.835	0.99 (0.70, 1.39)	0.954	0.99 (0.70, 1.41)	0.956

Charlson comorbidity index						
0	1 (ref)		1 (ref)		1 (ref)	
1	1.50 (1.48, 1.52)	<.001	1.59 (1.57, 1.62)	<.001	1.41 (1.39, 1.43)	<.001
≥ 2	2.09 (2.07, 2.12)	<.001	2.33 (2.28, 2.37)	<.001	1.86 (1.83, 1.90)	<.001
eGFR, decile						
1st	1.34 (1.31, 1.37)	<.001	1.39 (1.35, 1.43)	<.001	1.22 (1.18, 1.25)	<.001
2nd	1.25 (1.23, 1.28)	<.001	1.25 (1.21, 1.29)	<.001	1.17 (1.13, 1.20)	<.001
3rd	1.22 (1.19, 1.25)	<.001	1.23 (1.19, 1.27)	<.001	1.11 (1.08, 1.15)	<.001
4th	1.13 (1.11, 1.16)	<.001	1.11 (1.08, 1.15)	<.001	1.08 (1.05, 1.12)	<.001
5th	1 (ref)		1 (ref)		1 (ref)	
6th	1.24 (1.21, 1.27)	<.001	1.29 (1.25, 1.33)	<.001	1.02 (0.98, 1.05)	0.382
7th	1.20 (1.17, 1.23)	<.001	1.13 (1.1, 1.17)	<.001	1.19 (1.15, 1.23)	<.001
8th	1.11 (1.09, 1.14)	<.0001	1.02 (0.99, 1.06)	0.181	1.07 (1.03, 1.10)	<.001
9th	0.84 (0.82, 0.86)	<.001	0.73 (0.71, 0.76)	<.000	0.99 (0.96, 1.03)	0.588
10th	0.52 (0.50, 0.54)	<.001	0.41 (0.39, 0.43)	<.001	0.66 (0.64, 0.69)	<.001

The multivariable model was adjusted for sex, age, waist circumference, income levels, smoking, alcohol consumption, regular physical activity, proteinuria, hypertension, dyslipidemia, heart failure, myocardial infarction, valvular heart disease, cardiomyopathy, hyperthyroidism, congenital heart disease, and Charlson comorbidity index.

HR, hazard ratio; CI, confidence interval; Q, quartile; eGFR, Estimated Glomerular Filtration Rate.

Supplementary Table S9. BMI-specific comparative analysis for association of renal hyperfiltration (range) with diabetes mellitus

Variable	Population (N=2,294,362)		BMI Group < 25 (n=1,579,452)		BMI Group ≥ 25 (n=714,906)	
	HR CI (95%)	*p*-value	HR CI (95%)	*p*-value	HR CI (95%)	*p*-value
Sex						
Male	1 (ref)		1 (ref)		1 (ref)	
Female	1.07 (1.06, 1.09)	<.001	1.01 (0.99, 1.03)	0.448	1.21 (1.18, 1.23)	<.001
Age, years						
< 65	1 (ref)		1 (ref)		1 (ref)	
≥65	1.67 (1.65, 1.69)	<.001	1.89 (1.86, 1.93)	<.001	1.40 (1.38, 1.43)	<.001
Household income						
Q1, lowest	1 (ref)		1 (ref)		1 (ref)	
Q2	0.91 (0.90, 0.92)	<.001	0.91 (0.89, 0.93)	<.001	0.91 (0.90, 0.93)	<.001
Q3	0.92 (0.91, 0.94)	<.001	0.94 (0.93, 0.96)	<.001	0.91 (0.89, 0.93)	<.001
Q4, highest	0.99 (0.98, 1.01)	0.441	1.01 (0.99, 1.04)	0.234	0.98 (0.96, .1.00)	0.059
Smoking status						
Never	1 (ref)		1 (ref)		1 (ref)	
Former	1.16 (1.14, 1.18)	<.001	1.18 (1.15, 1.21)	<.001	1.14 (1.11, 1.17)	<.001
Current	1.19 (1.17, 1.21)	<.001	1.2 (1.18, 1.23)	<.001	1.16 (1.13, 1.19)	<.001
Alcohol consumption (days/week)						
None	1 (ref)		1 (ref)		1 (ref)	
1-4	0.82 (0.81, 0.83)	<.001	0.80 (0.79, 0.82)	<.001	0.84 (0.83, 0.86)	<.001
≥ 5	1.12 (1.1, 1.15)	<.001	1.18 (1.14, 1.22)	<.001	1.06 (1.03, 1.10)	<.001
Regular physical activity (days/week)						
None	1 (ref)		1 (ref)		1 (ref)	
1-4	1.02 (1.00, 1.04)	0.029	1.07 (1.04, 1.09)	<.001	0.98 (0.95, 1.00)	0.072
≥ 5	0.97 (0.96, 0.98)	<.001	1.00 (0.98, 1.02)	0.789	0.95 (0.93, 0.96)	<.001
Waist circumference (cm)	1.01 (1.01, 1.01)	<.001	1.01 (1.01, 1.01)	<.001	1.01 (1.01, 1.01)	<.001
Proteinuria						
Negative (-)	1 (ref)		1 (ref)		1 (ref)	
Positive (+)	1.31 (1.28, 1.34)	<.001	1.29 (1.25, 1.34)	<.001	1.34 (1.30, 1.38)	<.001
Comorbidities						
Hypertension	1.56 (1.54, 1.58)	<.001	1.66 (1.63, 1.69)	<.001	1.47 (1.45, 1.50)	<.001
Dyslipidemia	1.39 (1.37, 1.41)	<.0001	1.61 (1.57, 1.64)	<.001	1.22 (1.19, 1.24)	<.001
Heart failure	1.14 (1.10, 1.18)	<.001	1.13 (1.07, 1.19)	<.001	1.18 (1.12, 1.23)	<.001
Myocardial Infarction	1.38 (1.28, 1.48)	<.001	1.43 (1.29, 1.58)	<.001	1.35 (1.22, 1.49)	<.001
Valvular heart disease	1.06 (0.98, 1.16)	0.136	1.14 (1.03, 1.27)	0.015	0.94 (0.83, 1.07)	0.346
Cardiomyopathy	1.18	0.017	1.26	0.027	1.15	0.154

	(1.03, 1.36)		(1.03, 1.54)		(0.95, 1.39)	
Hyperthyroidism	1.30 (1.25, 1.36)	<.001	1.39 (1.32, 1.47)	<.001	1.20 (1.12, 1.27)	<.001
Congenital heart disease	0.96 (0.75, 1.23)	0.731	0.97 (0.69, 1.37)	0.859	0.98 (0.69, 1.40)	0.918
Charlson comorbidity index						
0	1 (ref)		1 (ref)		1 (ref)	
1	1.52 (1.51, 1.54)	<.001	1.62 (1.59, 1.65)	<.001	1.42 (1.40, 1.45)	<.001
≥ 2	2.14 (2.11, 2.17)	<.001	2.39 (2.34, 2.43)	<.001	1.89 (1.86, 1.93)	<.001
eGFR, range						
<30	1.66 (1.47, 1.89)	<.001	1.74 (1.47, 2.07)	<.001	1.59 (1.32, 1.92)	<.001
30-60	1.10 (1.07, 1.12)	<.001	1.13 (1.09, 1.16)	<.001	1.09 (1.05, 1.12)	<.001
60-90	1 (ref)		1 (ref)		1 (ref)	
90-120	0.88 (0.87, 0.89)	<.001	0.84 (0.82, 0.85)	<.001	0.92 (0.91, 0.94)	<.001
>120	0.40 (0.39, 0.42)	<.001	0.32 (0.30, 0.34)	<.001	0.56 (0.53, 0.59)	<.001

The multivariable model was adjusted for sex, age, waist circumference, income levels, smoking, alcohol consumption, regular physical activity, proteinuria, hypertension, dyslipidemia, heart failure, myocardial infarction, valvular heart disease, cardiomyopathy, hyperthyroidism, congenital heart disease, and Charlson comorbidity index.

HR, hazard ratio; CI, confidence interval; Q, quartile; eGFR, Estimated Glomerular Filtration Rate.
